# The association between maternal care access and term stillbirth at the US county level

**DOI:** 10.1002/pmf2.70225

**Published:** 2026-02-02

**Authors:** Isabella F. McNamara, Mohak Mhatre, Phinnara Has, Bianca Alonso-Bermudez, Sebastian Z. Ramos

**Affiliations:** 1 Division of Maternal Fetal Medicine, Department of Obstetrics and Gynecology, Tufts University School of Medicine, Boston, Massachusetts, USA; 2 Lifespan Biostatistics, Epidemiology, and Research Design, Rhode Island Hospital, Providence, Rhode Island, USA

**Keywords:** maternal care access, maternal care deserts, perinatal outcomes, rural care, stillbirth

## Abstract

**Introduction:**

Access to comprehensive maternal care is associated with a reduction in stillbirth rates. Given the decreasing access to maternal care across the United States, the aim of this study was to investigate the association of county-level maternal care access in the United States with stillbirth rate at term.

**Methods:**

This is a cross-sectional study using Centers for Disease Control and Prevention (CDC) Vital Statistics birth certificate data from 2016 to 2019. All non-anomalous, singleton births of ≥37 weeks’ gestation were included. Maternal care access level was defined by the 2020 March of Dimes Maternal Care Access Report county data that classified maternal care access as full, moderate, low, or desert, which they based on the availability of hospitals providing obstetric care, the number of obstetric providers per 10,000 births, and the percent of uninsured women. The primary outcome was stillbirth at term (≥37 weeks’ gestational age). Mixed-effects multivariable models were used and adjusted for clinical, demographic, and county-level factors, including access to community resources as defined by the CDC’s Social Vulnerability Index (SVI).

**Results:**

A total of 3138 counties were included in this analysis with 1100 (35.1%) of those counties located in maternal care deserts. Out of 13,304,743 births, term stillbirth occurred in 16,402 deliveries (0.12%) with 13.2% of these stillbirths occurring in counties without full access to maternal care. In unadjusted models, there were increased odds of stillbirth in maternal care deserts (odds ratio [OR], 1.13; 95% confidence interval [CI], 1.04–1.22) when compared to full maternal care access. However, this was not statistically significant after adjusting for individual and county-level factors (adjusted odds ratio [aOR], 0.98; 95% CI, 0.89–1.09).

**Conclusion:**

More than one third of US counties are in maternal care deserts and 13% of term stillbirths occur in areas without full access to maternal care. Although the association between county-level maternal care access and term stillbirth was attenuated after adjustment for individual and contextual factors, these findings highlight important geographic inequities in care availability. Continued work using more granular, patient-level and facility-level measures of access, including care modalities, care quality, and actual service utilization, is needed to better characterize the pathways through which declining obstetric care infrastructure may influence stillbirth risk.

## 1 | INTRODUCTION

Stillbirth is one of the most common adverse outcomes of pregnancy. In the United States, the Centers for Disease Control and Prevention (CDC) reports 21,000 stillbirths on an annual basis, corresponding to 5.73 fetal deaths per 1000 live births at 20 weeks of gestation or more [1]. The number of stillbirths worldwide has steadily decreased over the last century, until 2000, when the rate of decline slowed [2]. Compared to other countries, the United States has made less progress toward reducing the rates of stillbirth [3]. The development of stillbirth prevention bundles, aimed at targeting known modifiable risk factors of stillbirth [4], has been discussed as a mechanism to help improve stillbirth rates. One known modifiable risk factor of stillbirth is access to maternal care, which has been associated with a reduction in stillbirth rates [5, 6]. Alarmingly, maternal care access (MCA) is rapidly decreasing across the United States, largely due to the closing of maternal care units and the reorganization of the workforce [5, 7].

There are few studies that evaluate the effects of limited access to maternal care on perinatal mortality across the United States. Prior studies have explored how lack of adherence to prenatal care schedules can lead to increased risk of stillbirth, primarily in the setting of hypertensive disorders [5, 6]. However, these studies did not explore how care availability of obstetric care may have contributed to a lack of adherence. Moreover, rural communities with access to obstetric care have been shown to have improved perinatal outcomes [8]. While studies demonstrate a correlation between lack of obstetric providers and worse outcomes, specifically in rural settings, MCA is a more nuanced metric of understanding a county’s ability to care for pregnant residents [9]. MCA goes beyond designations of rural compared to urban location. Although many studies have explored individual determinants of stillbirth, less is known about how county-level access to maternal care services shapes stillbirth risk. In this study, we aim to understand the association between county-level MCA and stillbirth across the United States.

## 2 | MATERIALS AND METHODS

This is a cross-sectional analysis of singleton, non-anomalous fetuses at 37 weeks or greater gestational age identified in the CDC-restricted Vital Statistics birth certificate data between the years 2016 and 2019.

The predictor value of interest, MCA, was defined using the 2020 March of Dimes Maternal Care Desert annual report, which utilized US Health Resources and Services Administration (HRSA) and Area Health Resources Files data from 2019 to define access to care [10]. The March of Dimes report designates MCA level by the number of hospitals and birth centers providing obstetric care in each county, the number of providers (obstetricians and certified nurse midwives) per 10,000 births, and the proportion of women aged 18–64 without health insurance. They defined four designations of MCA including deserts, low access, moderate access, and full access to care (Box 1) [7]. Further, counties were considered urban (defined as >50,000 people residing in that county) or rural (defined as <50,000 people residing in that county) [11]. To accurately assess access to maternal care, we used the county of maternal residence rather than the county of delivery, as it better reflects the services available to a patient throughout pregnancy. This approach avoids misclassifying pregnant patients who travel from maternal care deserts to deliver in better-resourced areas and provides a more accurate measure of geographic barriers to care.

To better understand whether term stillbirth risk was due to higher levels of social disadvantage generally and not just access to maternal care, we controlled for the Social Vulnerability Index (SVI), a scale created by the CDC to measure social vulnerability at the county level [12]. The index includes 16 census variables of county-level factors, including socioeconomic status, racial/ethnic minority status, housing type, and transportation access. Prior studies have demonstrated an association between adverse pregnancy outcomes and the SVI [13]. We tested for potential collinearity between SVI and MCA by assessing the variance inflation factor (VIF) between these two variables after fitting a regression model with SVI as the outcome and MCA as the predictor. As a general guideline, a VIF of 10 or greater indicates potential collinearity that would need to be addressed.

The primary outcome was stillbirth diagnosed at 37 weeks or greater gestation. This study was limited to singleton, non-anomalous, term pregnancies to minimize potential fetal confounders as well as to apply a routine standard for all counties of being able to care for term pregnant patients. The analysis was carried out using mixed-effects logistic regression models to estimate the association between MCA level and stillbirth. The random effects structure of this analysis includes a random intercept by county to account for the lack of independence of individuals in each county. Two adjusted mixed-effects logistic regression models were included in this analysis, first adjusting for individual baseline characteristics including advanced maternal age (>35 years), body mass index (BMI) >30 kg/m^2^, pre-gestational diabetes, smoking, chronic hypertension, trimester of initiation of prenatal care, and county-level community vulnerability using the SVI and rurality. In the second model, the prior characteristics were included while also adjusting for pregnancy-specific risk factors including hypertensive disorders of pregnancy, gestational diabetes, smoking during pregnancy, and severe fetal growth restriction. Since fetal growth restriction is a prenatal diagnosis and not available through vital statistics data, birthweight was used as a representative variable. Severe small for gestational age (SGA) for female infants at less than the third percentile was defined by the Fenton Growth curve using 2100 g at 37 weeks and was included as a dichotomous variable. Female infant weight was used to ensure the inclusion of most of the severe fetal growth restriction cases [14]. A complete case analysis was conducted and those with missing variables were excluded.

Given the discrepancy in the dates of the fetal death data ranging from 2016 to 2019 and the use of the 2020 March of Dimes report, which relies on 2019 AHRF/HRSA data, a single sensitivity analysis was done for the year 2019 to ensure that no meaningful change in exposure classification was contributing to the study findings. Analyses were conducted using STATA/MP 18.0 (StataCorp LLC).

## 3 | RESULTS

There were 13,304,743 total births that met the inclusion criteria during the study period of 2016–2019. The majority of birthing patients resided in counties with full access to maternal care (88%), while 4% resided in maternal care deserts. There were a total of 3138 counties included, with 35.1% classified as maternal care deserts, 11.7% as low access, 6.8% as moderate access, and 46.4% as full access (Table 1). Maternal care deserts were predominantly in rural counties (88.8%), whereas only 5.3% of counties with full access to maternal care were in rural counties. Patients who lived in counties with full access to maternal care were more likely to be of advanced maternal age (18.2%), nulliparous (39.1%), and have more than a high school education (63.1%) compared to those that delivered in care deserts. Demographically, a larger proportion of patients who lived in maternal care deserts were identified as Non-Hispanic White (74.0%) than in full access areas (49.9%) (Table 1).

Stillbirth at term occurred in 16,402 (0.12%) births. Of these, 2166 (13.2%) term stillbirths occurred in counties without full access to maternal care. The mean gestational age of stillbirth in all counties was 38 weeks of gestational age and did not differ across MCA category. In unadjusted models, those who lived in a maternal care desert were more likely to experience stillbirth at term (odds ratio [OR], 1.13; 95% confidence interval [CI]; 1.04–1.22) (Table 2). However, in the mixed-effects multivariable models adjusting for known prepregnancy risk factors, the significance was not persistent (aOR 1.00, 95% CI 0.90–1.11). In the second adjusted model for both prepregnancy risk factors and those risk factors that develop during pregnancy (including hypertensive disorders of pregnancy, gestational diabetes, SGA, and smoking during pregnancy), MCA and stillbirth risk were not statistically significant (adjusted odds ratio [aOR] 0.98; 95% CI, 0.89–1.09). Finally, the VIF values for SVI and each level of MCA ranged from 1 to 4, indicating low likelihood that collinearity biased the study results. A sensitivity analysis was performed to only include the year 2019; results between data restricted to 2019 and overall study findings were similar (Table 3).

## 4 | DISCUSSION

Our findings demonstrate that a significant portion of stillbirths in the United States occur in counties without full access to maternal care. We found that the odds of stillbirth are significantly influenced by other known risk factors and not independently related to maternal access as measured by the March of Dimes designation. More specifically, we found that when controlling for factors related to prepregnancy health conditions, the association that was noted between MCA and stillbirth was no longer statistically significant. These results support focusing efforts on improving access to healthcare, particularly in rural areas, and optimizing health in the preconception period.

These findings are consistent with previously reported data demonstrating that stillbirth is an outcome that disproportionately impacts those living in rural settings and those with pre-existing risk factors [2, 5, 8]. Interestingly, our study was unable to elucidate the independent effect of MCA on the rate of stillbirth as measured by the March of Dimes access designation, suggesting that more granular data on access to care is needed to adequately determine the relationship between MCA in the United States and rates of stillbirth.

One of the strengths of this study was our ability to investigate this outcome at the county level. Both states and regions vary significantly in access to care, making county-level data an attractive unit of measure for understanding and thinking about solutions to the challenges of MCA. Additionally, by integrating the Social Vulnerability Index into the analysis, we adjusted for multiple community-level socioeconomic and environmental factors that could otherwise obscure the association between MCA and stillbirth risk.

Our study does have limitations. First, while county-level data were used as a robust measure to understand care available to individual patients within that county, not all counties without full access to maternal care have the same distance for travel to medical services or limitations in seeking care. Moreover, specific individuals’ access to care throughout pregnancy is a challenging measure to ascertain, given the limitations of categorical variables. The March of Dimes characterization of counties is meant to categorize complete counties and monitor changes in county-level access. The designated parameters may be insufficient to capture the influence that care access has on stillbirth risk at the population level. These limitations point to a need for more granular data to better understand how MCA can affect an individual’s risk of stillbirth. Moreover, the inability to exactly capture each county’s status in each year is a limitation of this study. The March of Dimes data are first available in the 2019 report that reflects 2018 data. Between the 2018 and 2019 data, 6% of counties shifted their access levels, with 3% increasing while 3% decreasing [7]. However, our sensitivity analysis that only included births in 2019 did not demonstrate differences in outcomes.

The use of birth certificate data is both a strength and limitation of this study. The use of the Vital Statistics Data allows for a large number of births to be included for what is an infrequent event, stillbirth at term. It allowed us to take into consideration the entire United States, which represents a large and diverse patient population that faces many unique challenges. However, there are limitations to the use of these data. Fetal death reports have been shown to have more missing data and more misclassification of data compared to standard birth certificates, which must be considered when interpreting the findings [15]. Additionally, this study was limited to singleton, non-anomalous, term pregnancies. Since higher order gestations, fetal anomalies, and earlier gestational age can all be associated with stillbirth, excluding these patients may have resulted in our study underestimating the association of adverse outcomes with MCA.

## 5 | CONCLUSION

In summary, MCA, as defined by the March of Dimes classification, was not independently associated with higher rates of term stillbirth after adjustment for individual-level risk factors. Nonetheless, a meaningful proportion of stillbirths occurred in counties with limited or no obstetric services, highlighting substantial geographic disparities in care availability. Future research should focus on more refined MCA and care quality to clarify the pathways through which the population-level maternal care environment influences stillbirth risk.

## Figures and Tables

**TABLE 1:** County-level characteristics, maternal demographics, and medical conditions.

	Total births (*n* = 13,304,743)	Maternal care desert (*n* = 535,410)	Low access to maternal care (*n* = 683,382)	Moderate access to maternal care (*n* = 366,659)	Full access to maternal care (*n* = 11,719,292)	*p* value
*County level characteristics*						
Births by year (%)						
2016	3,433,670 (25.8)	137,528 (25.7)	174,744 (25.6)	94,257 (25.7)	3,027,141 (25.8)	<0.001^a^
2017	3,345,886 (25.2)	134,302 (25.1)	170,832 (25.0)	91,899 (25.1)	2,948,853 (25.2)	
2018	3,289,265 (24.7)	132,726 (24.8)	169,360 (24.8)	90,708 (24.7)	2,896,471 (24.7)	
2019	3,235,922 (24.3)	130,854 (24.4)	168,446 (24.5)	89,795 (24.5)	2,846,827 (24.3)	
US counties (*n*)	3,138	1,100	367	214	1,457	<0.001^b^
Rural county (%)	1,535,506 (11.5)	475,591 (88.8)	278,800 (40.8)	161,700 (44.1)	619,415 (5.3)	<0.001^a^
Urban county (%)	11,769,237 (88.5)	59,819 (11.2)	404,582 (59.2)	204,959 (55.9)	11,099,877 (94.7)	
Social vulnerability index						
Mean (SD)	0.60 (0.26)	0.54 (0.28)	0.67 (0.24)	0.37 (0.24)	0.61 (0.26)	
Median (Q1–Q3)	0.65 (0.38–0.83)	0.56 (0.33–0.79)	0.71 (0.48–0.88)	0.32 (0.19–0.56)	0.66 (0.39–0.83)	
Range (min–max)	(0–1)	(0–0.99)	(0.01–0.99)	(0.001–0.93)	(0.0003–1)	<0.001^c^
*Stillbirth outcome*						
Stillbirths (%)	16,402 (0.12)	759 (0.14)	919 (0.13)	488 (0.13)	14,236 (0.12)	<0.001^a^
*Maternal demographics*						
Maternal age at delivery						
Mean (SD)	28.9 (5.8)	26.9 (5.5)	27.2 (5.6)	27.8 (5.4)	29.1 (5.7)	<0.001^c^
Median (Q1–Q3)	29 (25–33)	26 (23–31)	27 (23–31)	28 (24–31)	29 (25–33)	
Range (min–max)	(12–50)	(12–50)	(12–50)	(12–50)	(12–50)	
Advanced maternal age (%)	2,306,965 (17.3)	51,985 (9.7)	74,533 (10.9)	43,049 (11.7)	2,137,398 (18.2)	<0.001^a^
Nulliparous (%)	5,128,153 (38.7)	187,473 (35.1)	242,860 (35.7)	130,870 (35.9)	4,566,950 (39.1)	<0.001^a^
Race/ethnicity						
Non-Hispanic White	6,966,799 (52.4)	396,300 (74.0)	426,564 (62.4)	302,012 (82.4)	5,841,923 (49.9)	<0.001^a^
Non-Hispanic Black	1,804,008 (13.6)	52,691 (9.8)	83,586 (12.2)	23,192 (6.3)	1,644,539 (14.0)	
Hispanic	3,126,690 (23.5)	60,302 (11.3)	134,701 (19.7)	25,480 (7.0)	2,906,207 (24.8)	
Asian	872,892 (6.6)	3,595 (0.7)	9,939 (1.5)	4,884 (1.3)	854,474 (7.3)	
Other	415,497 (3.1)	20,978 (3.9)	26,338 (3.9)	9,211 (2.5)	358,970 (3.1)	
Unknown	118,857 (0.9)	1,544 (0.3)	2,254 (0.3)	1,880 (0.5)	113,179 (1.0)	
Maternal education						
Less than high school	1,666,024 (12.7)	78,087 (14.6)	111,614 (16.4)	41,117 (11.3)	1,435,206 (12.4)	
High school	3,329,199 (25.4)	177,825 (33.4)	216,673 (31.9)	105,638 (29.0)	2,829,063 (24.5)	
More than high school	8,136,258 (62.0)	277,302 (52.0)	351,780 (51.7)	217,626 (59.7)	7,289,550 (63.1)	<0.001^a^
Delivery BMI (kg/m^2^)						
Mean (SD)	26.9 (6.7)	28.1 (7.3)	27.8 (7.1)	27.8 (7.1)	26.9 (6.6)	
Median (Q1–Q3)	25.5 (22.1–30.4)	26.6 (22.7–32.1)	26.4 (22.6–31.7)	26.3 (22.6–31.7)	25.4 (22.1–30.2)	
Range (min–max)	(13–69.9)	(13.1–69.5)	(13–69.8)	(13.1–69.3)	(13–69.9)	<0.001^c^
*Prenatal care initiation*						
No prenatal care	189,574 (1.4)	7477 (1.4)	12,028 (1.8)	3,014 (0.8)	167,055 (1.4)	<0.001^a^
First trimester	10,147,830 (76.3)	397,579 (74.3)	490,213 (71.7)	288,746 (78.8)	8,971,292 (76.6)	
Second trimester	2,065,286 (15.5)	94,396 (17.6)	132,312 (19.4)	55,944 (15.3)	1,782,634 (15.2)	
Third trimester	585,759 (4.4)	25,813 (4.8)	35,241 (5.2)	13,914 (3.8)	510,791 (4.4)	
Unknown	316,014 (2.4)	10,143 (1.9)	13,581 (2.0)	5,036 (1.4)	287,254 (2.5)	
*Comorbid medical conditions*						
Pre-existing diabetes (%)	101,899 (0.77)	4,451 (0.83)	5,907 (0.87)	2,946 (0.80)	88,595 (0.76)	<0.001^a^
Gestational diabetes (%)	822,118 (6.2)	30,375 (5.7)	38,966 (5.7)	23,876 (6.5)	728,901 (6.2)	<0.001^a^
Chronic hypertension (%)	220,529 (1.7)	11,185 (2.1)	13,563 (1.9)	7,555 (2.1)	188,226 (1.6)	<0.001^a^
Pregnancy-induced hypertension (%)	774,522 (5.8)	35,007 (6.6)	43,224 (6.3)	25,911 (7.1)	670,380 (5.7)	<0.001^a^
Tobacco use in pregnancy (%)	837,047 (6.3)	76,196 (14.3)	72,988 (10.7)	54,129 (14.8)	633,734 (5.4)	<0.001^a^
Severe small for gestational age (%)	60,681 (0.46)	2885 (0.54)	3723 (0.55)	1562 (0.43)	52,511 (0.45)	<0.001^a^

*Note*: Categorical data are *n*(%).

Abbreviation: BMI, body mass index.

a Pearson’s chi-squared.

b Poisson regression.

c Kruskall–Wallis.

**TABLE 2:** Adjusted and unadjusted mixed effect models reflecting risk of stillbirth by level of maternal care access.

	Unadjusted model		Model 1^a^		Model 2^b^	
	Crude OR (95% CI)	*p* value	aOR (95% CI)^a^	*p* value	aOR (95% CI)^b^	*p* value
Total stillbirths						
Maternal care desert	1.13 (1.04–1.22)	0.003	1.00 (0.90–1.11)	0.99	0.98 (0.88–1.09)	0.75
Low access to maternal care	1.08 (0.99–1.16)	0.055	1.00 (0.92–1.09)	0.96	1.00 (0.92–1.09)	0.99
Moderate access to maternal care	1.07 (0.97–1.18)	0.19	1.08 (0.97–1.21)	0.15	1.09 (0.97–1.22)	0.14
Full access to maternal care	*Referent*	*Referent*	*Referent*	*Referent*	*Referent*	*Referent*

Abbreviations: aOR, adjusted odds ratio; BMI, body mass index; CI, confidence interval; OR, odds ratio; SGA, small for gestational age; SVI, Social Vulnerability Index.

a Model 1 adjusted for advanced maternal age (>35 years), BMI >30 kg/m^2^, education, prenatal care initiation, chronic hypertension, pregestational diabetes, rural county, and SVI.

b Model 2 adjusted for factors in Model 1 plus hypertensive disorders of pregnancy, Gestational diabetes (GDM), severe SGA, and smoking during pregnancy.

**TABLE 3:** Adjusted and unadjusted mixed effect models reflecting risk of stillbirth by level of maternal care access restricted to 2019.

	Unadjusted model		Model 1^a^		Model 2^b^	
	Crude OR (95% CI)	*p* value	aOR (95% CI)^a^	*p* value	aOR (95% CI)^b^	*p* value
Total stillbirths						
Maternal care desert	1.08 (0.92–1.27)	0.32	0.95 (0.79–1.16)	0.63	0.92 (0.76–1.12)	0.40
Low access to maternal care	1.09 (0.95–1.27)	0.22	0.98 (0.82–1.17)	0.81	0.96 (0.80–1.14)	0.62
Moderate access to maternal care	1.05 (0.86–1.28)	0.63	0.98 (0.79–1.22)	0.84	0.99 (0.79–1.25)	0.99
Full access to maternal care	*Referent*	*Referent*	*Referent*	*Referent*	*Referent*	*Referent*

Abbreviations: aOR, adjusted odds ratio; BMI, body mass index; CI, confidence interval; OR, odds ratio; SGA, small for gestational age; SVI, Social Vulnerability Index.

a Model 1 adjusted for advanced maternal age (>35 years), BMI >30 kg/m^2^, education, prenatal care initiation, chronic hypertension, pregestational diabetes, rural county, and SVI.

b Model 2 adjusted for factors in Model 1 plus hypertensive disorders of pregnancy, GDM, severe SGA, and smoking during pregnancy.

## Box

**BOX 1:** March of Dimes definition of maternal care deserts and access to maternal care.

Definition	Maternal care desert	Low access to maternal care	Moderate access to maternal care	Full access to maternal care
Hospitals and birth centers offering obstetric care	Zero	<2	<2	>2
Obstetric providers (obstetricians, CNM/CM) per 10,000 births	Zero	<60	<60	≥60
Proportion of women 18–64 without health insurance	Any percentage of women without health insurance	≥10%	<10%	Any percentage of women without health insurance

Abbreviations: CM, certified midwife; CNM, certified nurse midwife.
